# Williams-Beuren syndrome in pediatric T-cell acute lymphoblastic leukemia: A rare case report and review of literature

**DOI:** 10.1097/MD.0000000000036976

**Published:** 2024-02-16

**Authors:** Rong Yang, Yuan Ai, Ting Bai, Xiao-Xi Lu, Guoqian He

**Affiliations:** a Key Laboratory of Birth Defects and Related Diseases of Women and Children, Ministry of Education, West China Second University Hospital, Sichuan University, Chengdu, Sichuan, P.R. China; b Department of Pediatrics, West China Second University Hospital, Sichuan University, Chengdu, Sichuan, P.R. China; c Department of Obstetrics & Gynecology, West China Second University Hospital, Sichuan University, Chengdu, Sichuan, P.R. China.

**Keywords:** 7q11.23, acute lymphoblastic leukemia, chromosomal microarray, pediatric leukemia, Williams-Beuren syndrome

## Abstract

**Background:**

Williams-Beuren syndrome (WBS) is a rare genetic disorder caused by hemizygous microdeletion of contiguous genes on chromosome 7q11.23. Although the phenotype features extensive heterogeneity in severity and performance, WBS is not considered to be a predisposing factor for cancer development. Currently, hematologic cancers, mainly Burkitt lymphoma, are rarely reported in patients with WBS. Here in, we report a unique case of T-cell acute lymphoblastic leukemia in a male child with WBS.

**Methods:**

This retrospective study analyzed the clinical data of this case receiving chemotherapy were analyzed. This is a retrospective study.

**Results:**

The patient, who exhibited a typical WBS phenotype and presented with hemorrhagic spots. Chromosomal genome-wide chip analysis (CMA) revealed abnormalities on chromosomes 7 and 9. The fusion gene *STIL-TAL1* and mutations in *BCL11B, NOTCH1*, and *USP7* have also been found and all been associated with the occurrence of T-cell leukemia. The patient responded well to the chemotherapy.

**Conclusion:**

To the best of our knowledge, this is the first reported case of WBS in T-cell acute lymphoblastic leukemia. We want to emphasize that the occurrence of leukemia in this patient might be related to the loss of 7q11.23 and microdeletion of 9p21.3 (including 3 TSGs), but the relationship between WBS and malignancy remains unclear. Further studies are required to clarify the relationship between WBS and malignancy.

## 1. Introduction

Williams-Beuren syndrome (WBS), also known as Williams syndrome, is a rare complex developmental disorder affecting multiple systems.^[1,2]^ The incidence of this disorder is approximately 1:10,000 live births, worldwide. WBS is a contiguous gene syndrome induced by a heterozygous deletion of 26 to 28 genes in the chromosome region 7q11.23.^[2–4]^ This syndrome is caused by an unbalanced recombination on chromosome 7 during meiosis. Most cases are detected using fluorescence in situ hybridization. The critical region encodes the elastin (*ELN*) gene, which contributes to some of the characteristic facial features of WBS.^[4,5]^

WBS is characterized by highly variable phenotypes.^[6,7]^ Patients usually show the characteristic “elfin face” appearance, growth retardation, “friendly” personality, cardiovascular abnormalities, intellectual disability, infantile hypercalcemia, connective tissue involvement, central nervous system involvement, and other clinical features.^[8–11]^ Children usually have early language delays and later develop relative strengths in language and auditory memory. Older children may also develop progressive joints disorders. Although WBS does not predispose carriers to cancer, there have been single reports of non-Hodgkin lymphoma and 1 case report of acute lymphoblastic leukemia (ALL) in children with WBS.^[12–20]^ Most ALL cases have some degree of karyotypic instability and congenital chromosomal abnormalities that persist in leukemic cells.

To the best of our knowledge, only a few cases of malignancies in patients with WBS have been published in hematologic malignancies and solid tumors, and only 1 young male child with WBS who was diagnosed with severe chronic kidney disease confirmed the presence of a T-cell lymphoblastic lymphoma after receiving GH replacement therapy.^[14]^ Herein, we present a rare case of T-cell ALL (T-ALL) in a child with WBS. Our study also identified other mutations and chromosomal abnormalities; however, the patient responded well to the CCCG-ALL-2020 chemotherapy.

## 2. Case

The patient was the second child of nonconsanguineous parents, born at term (38 weeks), weighing 2400 g (<2nd percentile), with a length of 45 cm (3rd percentile), and normal head circumference. The patient was diagnosed at an early gestational age. The mother had previously presented with hypothyroidism and a spontaneous abortion. The patient father was in apparent good health. Pregnancy was not complicated by hypoxia. Prenatal ultrasonography revealed that the baby had a slightly smaller biparietal diameter. However, the mother did not receive regular antenatal care. Growth retardation and a left inguinal hernia were diagnosed during infancy. The patient had a small head circumference, was unable to raise his neck at 5 months of age, and had a delay in psychomotor development and poor growth since 5 months of age. Brain magnetic resonance imaging (781–296Achieva 1.5T Nova Dual HP, Philips) showed no structural abnormalities. Biochemical laboratory evaluations were non-diagnostic. For economic reasons, the patient did not receive regular health services for children or undergo further examinations. The patient underwent rehabilitation training in the outpatient department and visited the neurology clinic; however, no further tests were performed. The patient was able to sit by himself at 11 months, stand alone at 18 months, and walk at 24 months. At follow-up, the patient was monosyllabic for 3 years.

We encountered the patient for the first time at the age of 5 years. He was initially treated for 8 days for acute hemorrhagic spots on the head, neck, and face. On admission, he had a typical elfin face, ocular hypertelorism, low-set, protruding ears, microcephalus, head circumference of 48 cm (<3rd percentile), height of 105 cm (<1st percentile), weight of 14.5 kg (<2nd percentile) (Fig. S1A, http://links.lww.com/MD/L742). In addition, the head shape was abnormal, with an anteroposterior dimension of 17 cm and a mediolateral dimension of 12 cm. The patient was slightly pale, and hemorrhagic spots were visible on the skin, along with lymphadenectasis of the neck and submandibular space. Hepatomegaly (2 cm), splenomegaly (4 cm), and a left inguinal hernia were present.

The Laboratory test results were abnormal. Routine blood examinations revealed a white blood cells 84.1 × 10^9^/L; L, 18%; N, 4%; hemoglobin, 81 g/L; platelets, 12 × 10^9^/L; and abnormal cells were detected (78%). Lactate dehydrogenase levels were increased (1910 IU/L). Blood Epstein-Barr virus, cytomegalovirus, TORCH screening, and blood transfusion immunoassays were normal. Serum vitamin D levels were also low. No hypercalcemia was not observed. Thyroid hormone tests suggested that the boy had low serum T3 at 1.05 nmol/L (1.29–3.0 nmol/L) and FT3 at 3.4 pmol/L (5.1–8.0 pmol/L) levels. No cardiovascular complications were observed in this patient. Before chemotherapy, a chest computed tomography (CT, Neuviz 128, Shenyang Neusoft Medical System Co., LTD) scan showed a negative result. Abdominal CT revealed splenic enlargement. The head CT and magnetic resonance imaging showed a “navicular” change to the shape of the skull, a small capsule shadow on the right bridge cerebellum horn pool, and minor signal abnormalities in bilateral ventricular white matter. The cerebrospinal fluid test results were normal results. Electroencephalography showed that the background waves were slightly slower than those of their peers, which might be related to the abnormal development of the nervous system in this child. Bone marrow cytological analysis before therapy revealed ALL (L2). This was confirmed by flow cytometry, which showed a monoclonal T-cell population expressing cCD3, CD3, CD7, CD4, CD8, CD1a, CD2, CD5, TDT, and CD38, and negative for CD19, cCD79a, cCD22, and MPO (Fig. S2, http://links.lww.com/MD/L743).

**Figure 1.**
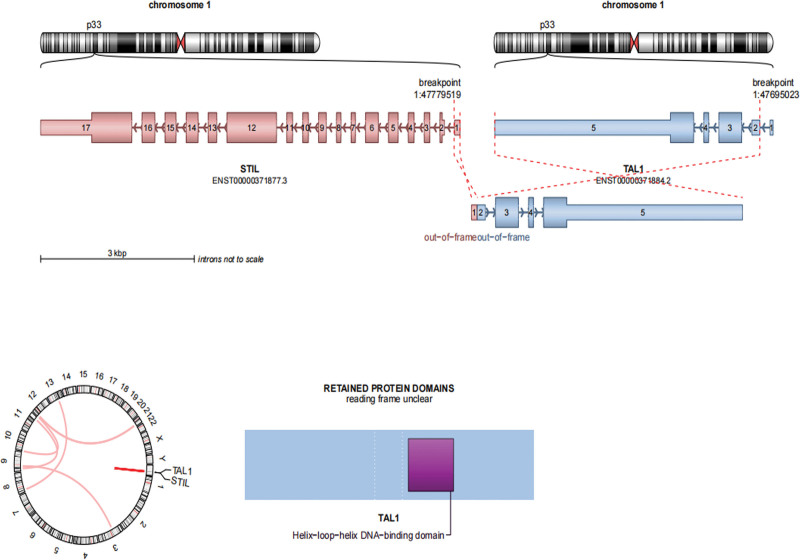
Whole transcriptome sequencing test found *STIL-TAL1* fusion gene positive.

**Figure 2.**
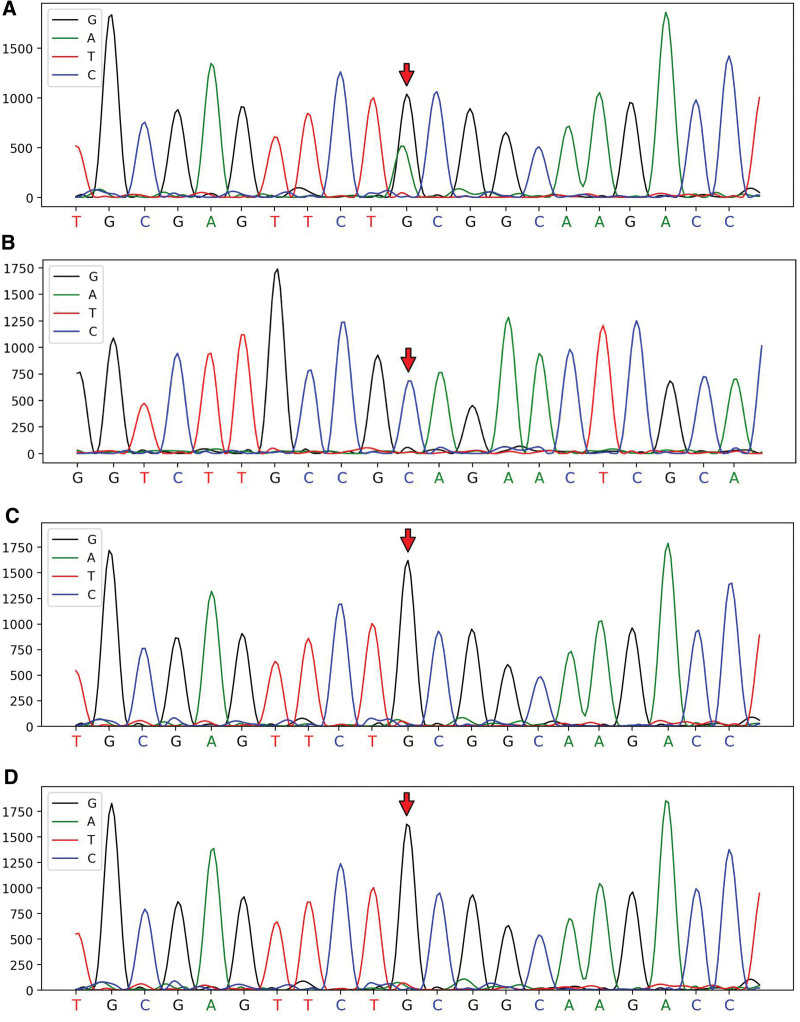
(A) The mutation of *BCL11B* gene in our patient was a heterozygous missense mutation and was confirmed as a de novo somatic mutation. (B) Heterozygous mutation of *BCL11B*, c.1295G > A in chr14.99641878. The wild type of hair in chr.14.99641878. (C) Wild type in chr.14.99641878 of the patient father. (D) Wild type in chr.14.99641878 of the patient mother.

Cytogenetic analysis revealed an 46, XY karyotype. However, chromosome genome-wide chip analysis (CMA) detected 2 different chromosomal abnormalities on chromosomes 7 and 9 (Fig. S1B, http://links.lww.com/MD/L742). Whole transcriptome (Illumina Nova Seq 6000 sequencer) and whole exome sequencing (NOVO Seq 6000 sequencer) revealed 1 fusion gene and 3 mutations associated with ALL. The *STIL-TAL1* fusion gene was positive, which could help diagnose T-ALL (Fig. 1). *NOTCH1* and *USP7* are frameshift variants that are clearly associated with ALL, whereas *BCL11B* is a missense variant that may be associated with ALL. *BCL11B* had a heterozygous missense mutation and was confirmed to be a de novo somatic mutation (Fig. 2A and B). There was a wild type in chr.14.99641878 from his father and mother (Fig. 2C and D).

**Figure 3.**
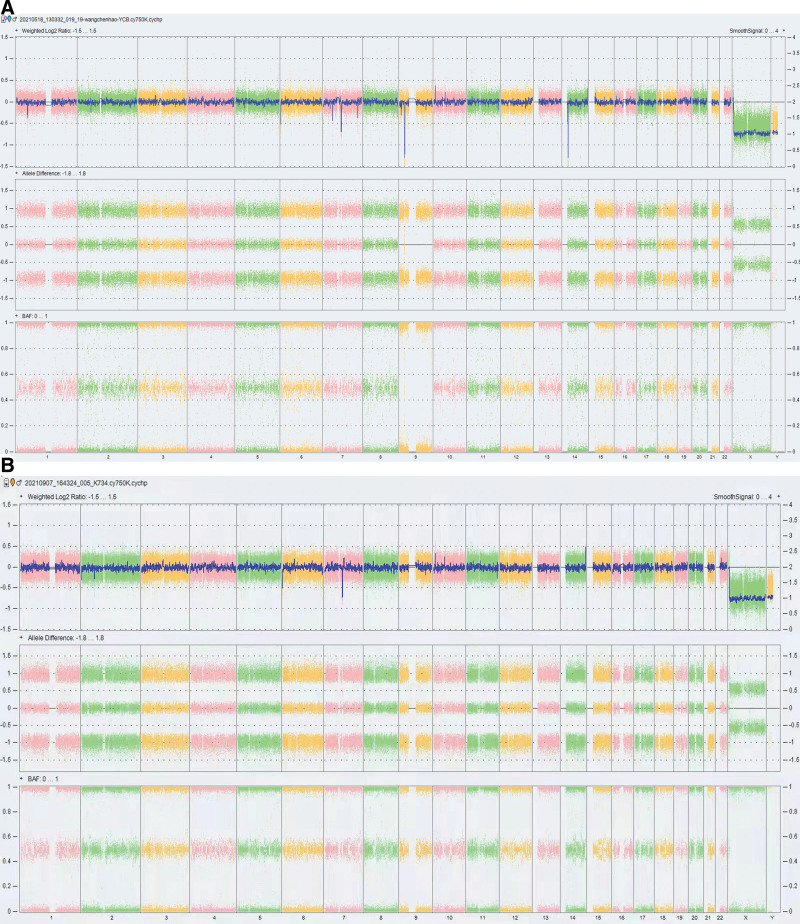
(A) Chromosomal microarray of peripheric blood found 2 pathogenic copy number variations (CNVs) and 2 chromosome aberrations with ambiguous clinical significance. (B) After the patient entered complete remission, chromosomal microarray of peripheric blood showed the microdeletion of 7q11.23 was still positive, while the other chromosome aberrations, loss of 9p21.3, loss of 7q34 and ROH 9p24.3 q34.3, all turned negative.

**Figure 4.**
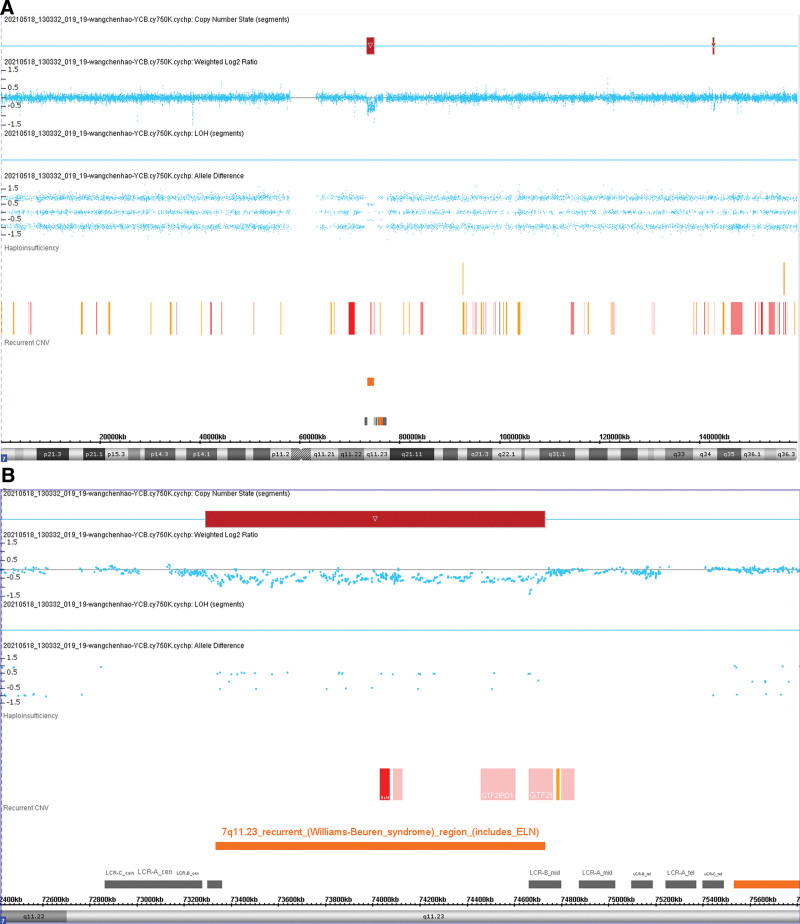
(A) Loss of 7q11.23 (1.44Mb). (B) Details of loss of 7q11.23 (ELN included). ELN = elastin.

CMA analysis of peripheral blood revealed 2 pathogenic copy number variations (CNVs) (Fig. 3A): loss of 7q11.23 (1.44 Mb, including ELN) (Fig. 4A and B) and loss of 9p21.3 (1.72 Mb) (Fig. S3A, http://links.lww.com/MD/L744), and 2 chromosomal aberrations with ambiguous clinical significance: loss of 7q34 (290 kb) (Fig. S4, http://links.lww.com/MD/L745) and region of homozygosity 9p24.3q34.3 (106.51 Mb) (Fig. S3B, http://links.lww.com/MD/L744). CMA analysis of the patient parents revealed no pathogenic CNVs, loss of heterozygosity, or uniparental disomy, indicating that all chromosomal aberrations were de novo. The CMA, PCR, FCM, cytogenetic analysis and fluorescence in situ hybridization were obtained from Kindstar Globalgene Technology Inc. (CCCG130152).

The patient was stratified into an intermediate-risk group and successfully treated for approximately 18 months with chemotherapy according to the Chinese Children Cancer Group 2020 protocol (CCCG-ALL-2020). After the first course of induction chemotherapy, the patient developed severe neutropenia and fever. Due to positive blood cultures for *Escherichia coli*, antibiotic therapy with imipenem/cilastatin was administered. Given the possibility of opportunistic fungal pathogen infiltration, supported by chest CT, the antifungal therapy was changed to voriconazole. On days 19 and 46 (calculated from the start of chemotherapy), a bone marrow smear suggested complete remission, and bone marrow aspiration and minimal residual disease was negative (<0.01%). The *STIL-TAL1* fusion gene and *NOTCH1, USP7*, and *BCL11B* mutations were absent. The peripheral blood was still positive for the microdeletion of 7q11.23, but the other chromosomal aberrations (loss of 9p21.3, loss of 7q34, and region of homozygosity 9p24.3q34.3) were all negative, indicating that the 7q11.23 microdeletion was a germline mutation, whereas the other chromosomal aberrations were somatic (Fig. 3B). The patient underwent rehabilitation training, which resulted in improved language, motor, and intellectual skills.

## 3. Discussion

WBS (OMIM 194050) was first described by Williams and Beuren in the 1960s and is characterized by unusual facial features, idiopathic hypercalcemia, supravalvular aortic stenosis, heart murmurs, and hypertension.^[21]^ WBS is considered an autosomal dominant genetic disorder, now known to be caused by a < 2 Mb heterozygous microdeletion of the WBS chromosomal region at 7q11.23.^[3,22,23]^ *ELN, GTF2I*, and *GTF2IRD1* genes in this region have been definitively linked to the key phenotypes of WBS.^[7,24–26]^ According to the published literature, the relationship between WBS and malignancy remains unclear. There have been limited reports on WBS in children with tumors, mostly blood cancers (Table 1). To the best of our knowledge, this is the first reported case of T-ALL in a patient who underwent WBS.

**Table 1:** Case reports of hematopoietic tumors in WBS patients of children.

Type of Hematopoietic malignancy	Yr	Age (yr)	Sex	Ref
ALL	2002	14	Male	^[13]^
NHL (Burkitt)	2004	8	Male	^[20]^
NHL (Burkitt)	2005	5	Female	^[5]^
NHL (T)	2009	12	Male	^[14]^
NHL (Burkitt)	2011	10	Female	^[19]^
Mature B-ALL	2013	8	Male	^[18]^
NHL (Burkitt)	2014	8	Female	^[17]^
B-NHL stage IV	2014	10	Male	^[17]^
NHL (Burkitt) with secondary malignancy (Ewing sarcoma)	2014	9	Unknown	^[16]^
Mature B-ALL	2016	10	Male	^[12]^
T-ALL	2022	5	Male	

ALL = acute lymphoblastic leukemia, NHL = non-Hodgkin lymphoma, T-ALL = T-cell acute lymphoblastic leukemia, WBS = Williams-Beuren syndrome.

In the present case, CMA detected 2 pathogenic de novo CNVs: loss of 7q11.23 and loss of 9p21.3. The microdeletion of 7q11.23 is considered a pathogenic CNV in WBS, and the lost segment contained the *ELN* gene, which is a haploinsufficiency gene with a score of 3 in the Clinical Genome Resource. This confirmed the diagnosis of WBS in the patient. However, the patient parents did not harbor CNVs, indicating that they were acquired de novo. To date, there is no evidence suggesting that the microdeletion of 7q11.23 is a tumor trigger. However, deletion of 9p21.3 is considered an unfavorable chromosomal aberration in renal cell carcinoma.^[27]^ There are at least 3 TSGs at 9p21.3: *CDKN2A, CDKN2B*, and *MTAP*, and deletion of 9p21.3 may predispose an individual to melanoma, neural system tumors, hematologic malignancies, astrocytoma, breast cancer, and chordoma.^[28–30]^ All 3 TSGs were included in the lost segment in the current patient, suggesting that the somatic microdeletion of 9p21.3 may have contributed to the occurrence of malignancy in this case. Somatic microdeletion of 9p21.3 was also reported in a patient with WBS with lymphoma and a homozygous deletion of the *INK4a/ARF* (or *CDKN2A*) locus at 9p21.3^[17]^; however, the relationship between 9p21.3 deletion and lymphoma has not been explained. The role of haploinsufficiency of genes located at 7q11.23 and the role of TSGs at 9p21.3 needs further investigation.

*BCL11B*, a member of the BCL family, plays a crucial role in the development, proliferation, differentiation, and subsequent survival of T cells.^[31]^ *BCL11B* encodes a zinc-finger transcription factor involved in hematopoietic progenitor cell development.^[32]^ *BCL11B* is hemizygously inactivated in approximately 10% of human T-cell ALLs,^[33]^ and the *BCL11B C432Y* mutation detected in this case has been previously reported in T-ALL.^[34]^ Mutations in *BCL11B* have also been reported in immunodeficiency 49 (OMIM 617237) and intellectual developmental disorders with dysmorphic facies, speech delays, and T-cell abnormalities (OMIM 618092), both of which exhibit autosomal dominant inheritance. *BCL11B* regulates neuronal differentiation and function during central nervous system development by controlling the proliferation and differentiation of glial progenitors.^[28,35]^ The *BCL11B* mutation in this patient was a heterozygous missense mutation, which was confirmed to be a de novo somatic mutation. However, the *BCL11B* mutation was not detected after chemotherapy, indicating that this mutation was related to leukemia but not to growth retardation in this patient. *NOTCH1* mutations have been identified in approximately 50% of pediatric T-ALL cases and predict a more rapid early treatment response and favorable long-term outcome.^[36,37]^ *USP7* cooperates with *NOTCH1* to drive oncogenic transcriptional programs in T-cell leukemia.^[38]^ Inhibition of *USP7* results in degradation of the oncogenic E3 ligase MDM2 and leads to reactivation of the tumor suppressor P53 in various cancers.^[39,40]^ The *STIL-TAL1* fusion gene is present in approximately 11% to 27% of pediatric T-cell ALLs and gives rise to inappropriate expression of *TAL1*, which may promote T-cell leukemogenesis.^[41]^

In addition, CMA of the peripheral blood revealed another chromosomal aberration with ambiguous clinical significance: loss of 7q34. However, the loss of 7q34 was not detected after chemotherapy remission in this patient. Therefore, the loss of 7q34 in this patient was due to an acquired chromosome copy number variation. It has been reported that the deletion of 7q34 is a recurrent chromosome abnormality in T-ALL patients. The main reason for this is that the deletion of 7q34 leads to an abnormal deletion of *TRB* gene expression.^[42]^ To the best of our knowledge, very few cases of WBS combined with tumors have been reported. Only 12 cases of WBS have been reported in combination with lymphatic hematopoietic system tumors, 8 cases with other solid tumors, and only 1 case with T-LBL. To the best of our knowledge, this is the first reported case of WBS concomitant with T-cell ALL.

## 4. Conclusion

In summary, we present the first report of a patient with WBS in T-ALL and WBS. The occurrence of leukemia in this patient might be related to the microdeletion of 7q11.23 and 9p21.3 (including the 3 TSGs). The fusion gene *STIL-TAL1* and mutations in *BCL11B, NOTCH1*, and *USP7* have been associated with T-cell leukemia. Further studies are required to clarify the relationship between WBS and malignancy.

## Acknowledgments

We thank the patient and her family for their assistance in this study.

## Author contributions

**Data curation:** Rong Yang, Ting Bai, Guoqian He.

**Investigation:** Ting Bai.

**Resources:** Yuan Ai.

**Supervision:** Xiao-Xi Lu, Guoqian He.

**Writing – original draft:** Rong Yang.

**Writing – review & editing:** Xiao-Xi Lu, Guoqian He.

## Supplementary Material








