# Supplementary figures and images for: Williams-Beuren syndrome in pediatric T-cell acute lymphoblastic leukemia: A rare case report and review of literature
Source: Medicine (Baltimore). 2024 Feb 16;103(7):e36976. doi: 10.1097/MD.0000000000036976 (PMC10869033; doi:10.1097/MD.0000000000036976)

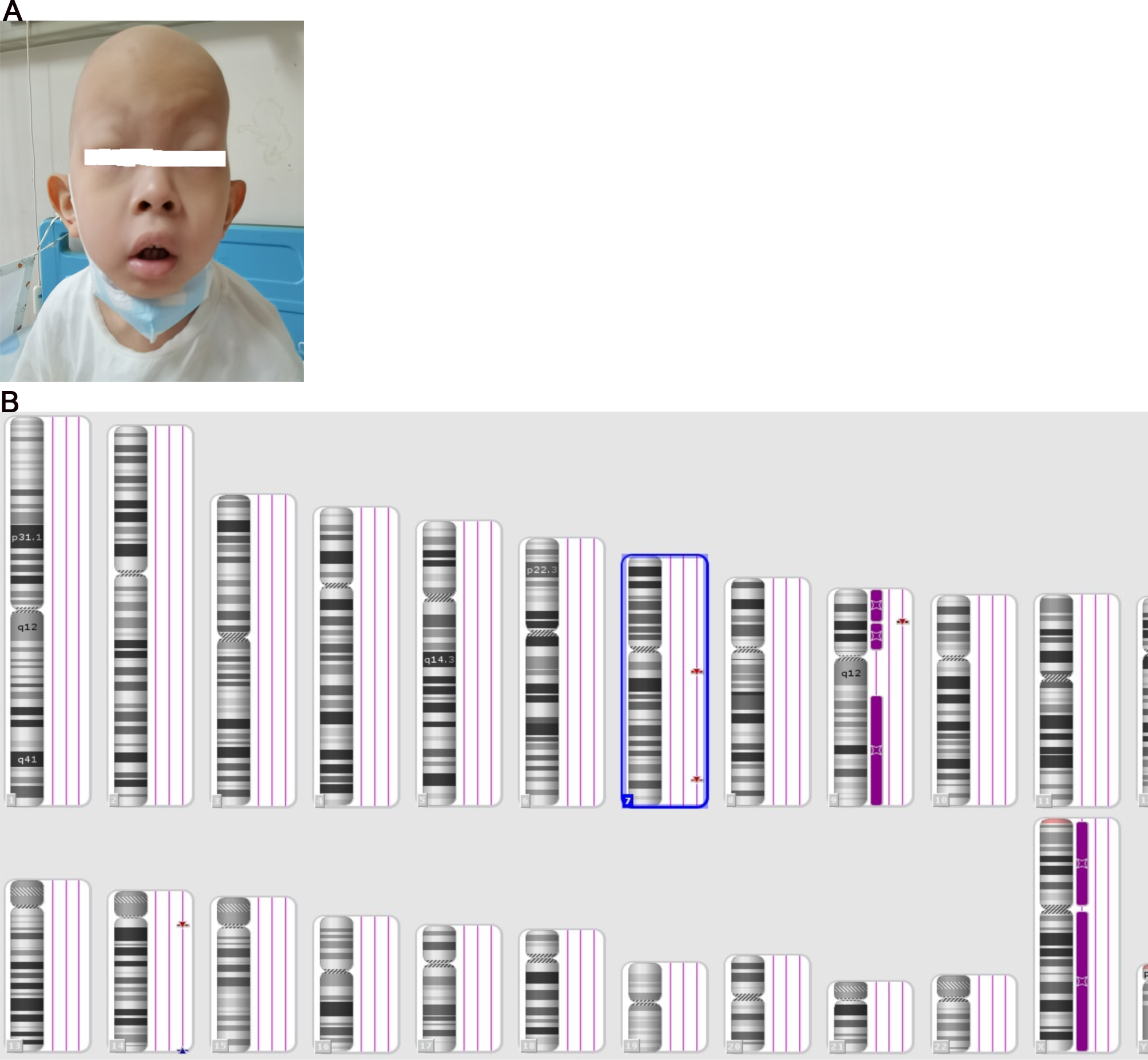

Supplement: Supplementary file 1 [file medi-103-e36976-s001.tif]

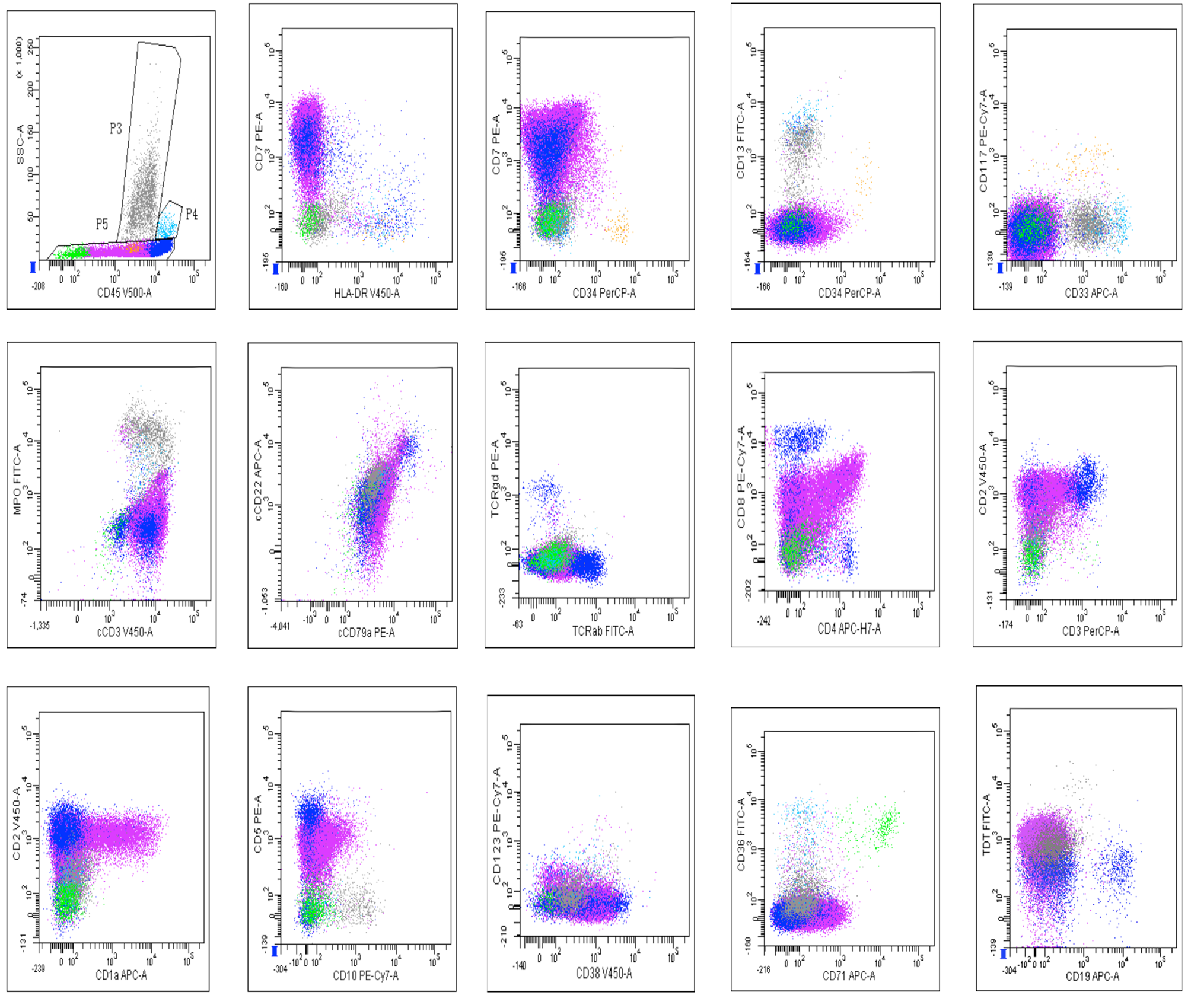

Supplement: Supplementary file 2 [file medi-103-e36976-s002.tif]

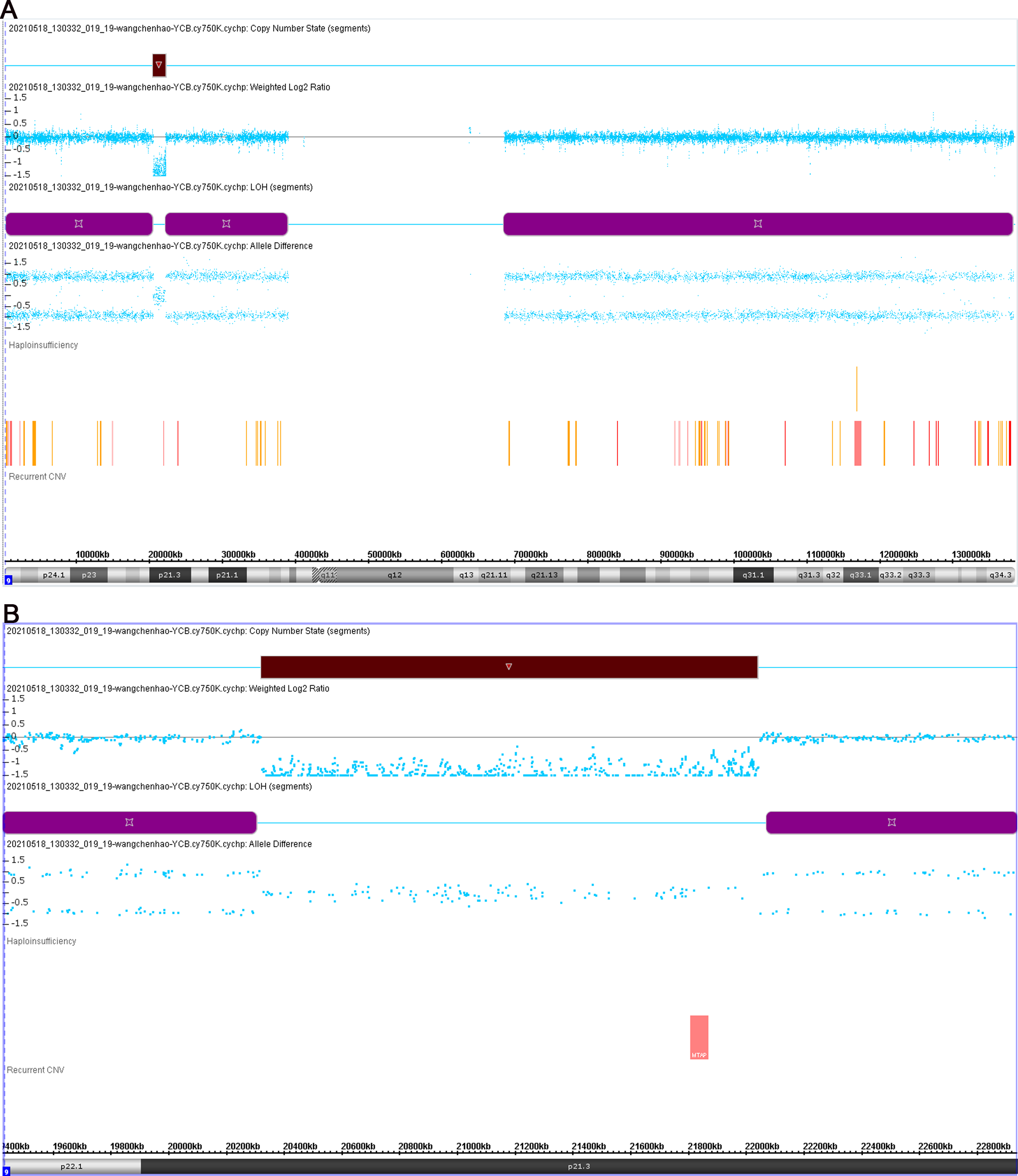

Supplement: Supplementary file 3 [file medi-103-e36976-s003.tif]
